# Medication adherence and factors associated with poor adherence among type 2 diabetes mellitus patients on follow-up at Kenyatta National Hospital, Kenya

**DOI:** 10.11604/pamj.2018.29.82.12639

**Published:** 2018-01-29

**Authors:** Gabriel Waari, Joseph Mutai, Joseph Gikunju

**Affiliations:** 1 School of Public Health, Institute of Tropical Medicine and Infectious Diseases, Jomo Kenyatta University of Agriculture and Technology, Kenya; 2 Centre for Public Health Research, Kenya Medical Research Institute; 3 Department of Medical Laboratory Science, Institute of Tropical Medicine and Infectious Diseases, Jomo Kenyatta University of Agriculture and Technology, Kenya

**Keywords:** Medication adherence, glycaemic control, type 2 diabetes

## Abstract

**Introduction:**

Medication non-adherence is a common problem facing health care providers treating adult type 2 diabetes mellitus patients. Poor glycaemic control associated with increased morbidity and mortality are resulting consequences. The objective of this study was to assess medication adherence among Type 2 diabetes mellitus patients.

**Methods:**

This is a cross-sectional study conducted at Kenyatta National Hospital from November 2015 to January 2016. 290 Type 2 diabetic patients were enrolled. A questionnaire was used for data collection. Adherence levels were determined by patient scores on Morisky Medication Adherence Scale-8 and glycaemic control by blood assay for glycosylated haemoglobin. Ordinal logistic regression modelling was done using STATA software to determine factors associated with poor medication adherence

**Results:**

The prevalence of medication adherence low for 28.3 % [95% CI: 23.1, 33.5], medium for 26.2% (95% CI: 21.1, 31.3) and high for 45.5% (95% CI: 39.6, 51.3) of study participants. Glycaemic control was good (HbA1c < 7%) for 107 (36.9 %) of study participants. Dissatisfaction with family members support (OR = 2.99, CI = 1.12-7.98), patients with 2-10 years duration of disease (OR = 2.07, CI = 1.01-4.22), ever being admitted for diabetes mellitus (OR = 2.94, CI = 1.60-5.41), challenge in drug access (OR = 1.76, CI = 1.01-3.05) and dissatisfaction with attending clinicians (OR = 3.58, CI= 1.36 - 9.43) were factors found associated with poor medication adherence.

**Conclusion:**

A majority of type 2 diabetes mellitus patients have suboptimal medication adherence. Family support, affordability of medications and good healthcare provider-patient communication are important in ensuring medication adherence.

## Introduction

Poor and inadequate glycaemic control among the patients with Type 2 diabetes mellitus (DM) constitutes a major public health problem and accelerates the development of diabetes compli-cations [1, 2]. In 2003, the World Health Organization (WHO) launched a landmark report which clearly defined “adherence” as the extent to which a person's behavior including taking medication corresponded to agreed recommendations from a health care provider. Secondly it recognized suboptimal medication adherence as a major factor leading to poor glycemic control among diabetic patients [3]. A variety of studies have continued to show that patients who fail to adhere to the prescribed clinical regimens have poorer outcomes including higher rates of complications [4,5]. Various models have been postulated to help understand barriers towards medication adherence [3, 6, 7]. All these models have common elements of factors that relate to the patient including socio-economic characteristics, factors that relate to the disease stage and treatment and factors that relate to the health system including clinician communication. Currently in sub-Saharan Africa, the International Diabetes Federation (IDF) estimates that there are about 14.2 million people living with diabetes; it is projected that in 2040 this number will increase to 34.2 million people [8]. In Kenya, the prevalence of DM was 3.3% in 2007 and is projected to get to 4.5% in 2025 [9]. The prevalence in some urban areas has been estimated to be up to 10% [10]. Chronic complications occur in significant proportions among Kenyan patients with both early and long-standing Type 2 DM and these are related to poor glycaemic control [11, 12]. However, the status of Type 2 DM patients' adherence to anti diabetic therapy and the factors associated with poor adherence to diabetic medication are yet to be adequately studied in Kenya. This study thus estimates the magnitude of medication adherence and secondly identifies some factors that are associated with poor adherence to medication among typical Kenyan Type 2 DM patients. This information will assist individuals with diabetes and their health care providers plan appropriate interventions to ensure optimal health outcomes.

## Methods

This was a cross-sectional study and was conducted from November 2015 and January 2016, at Kenyatta National Hospital. This is the largest public referral and teaching hospital in Kenya. The study population comprised of Type 2 diabetes mellitus patients (males and females) enrolled at the diabetes clinic and on oral or injection medication or both. The study participants had to be over 18 years and enrolled at the clinic for at least one month. Patients excluded for the study were patients attending their first visit, those aged below 18 years, Type 1 diabetes mellitus patients and those who were seriously ill or unable to speak. Sample size was determined using single proportion formula [13] considering a 95% confidence level, 5% margin of error and a known prevalence of adherence to diabetic medication among Type 2 diabetes mellitus patients of 25% [14]. This resulted in a calculated sample size of 289 patients. Study participants were selected using a systematic sampling procedure. On each day of the study, the first participant was determined by writing down the names of the first two patients in separate papers and thereafter choosing one randomly. Thereafter, every other patient that meets the selection criteria was enrolled into the study.

**Data collection**: A structured questionnaire was used to gather information regarding socio-demographic characteristics. The participant's clinic file was reviewed to obtain information regarding the medication regimen and known co-morbid/complication states. Evaluation of medication adherence was done using the Morisky Medication Adherence Scale-8 (MMAS-8) [15-17]. A score of 8 indicates high adherence, a score of 6-7 indicates medium adherence whilst a score of less than 6 indicates poor adherence. Alcoholism screening was done using the CAGE test [18]. Item responses on the four-item CAGE test are scored 0 or 1; with a higher score an indication of alcohol problems. The study participants had two anthropometric measurements taken. Height was be measured without shoes to the nearest of 0.1 centimeter (cm) using a stand-meter. Weight was measured to the nearest of 0.1 kg on a hospital scale, with the participant wearing one-layer of clothes and with no shoes. Body Mass Index (BMI) was calculated as weight (kg) divided by the square of the height (m^2^). The cut-offs for BMI were be based on the WHO criteria, where underweight is defined as BMI < 18.5 kg/m^2^, normal weight as BMI between 18.5 kg/m^2^ and 24.99 kg/m^2^, over weight is defined as a BMI ≥ 25 kg/m^2^ and obesity is defined as a BMI ≥ 30 kg/m^2^ [19]. Finger pin-prick blood assay of glycosylated hemoglobin (HbA1C) was also done using the A1cNow^®^ (PTS Diagnostics, IN, USA) point of care system.

**Data analysis**: Analysis of the collected data was performed using STATA version 11.0 statistical software. Descriptive statistics such means, proportions and frequencies were used to express participants socio-demographic, clinical and anthropometric characteristics. Medication adherence prevalence was determined by proportion of patients who obtain a score of 8 on the Morisky Medication Adherence Scale. HbA1C values determined by blood assay were categorized to either good control for patients whose values were less than 7% and poor control for patients whose values were 7% or more. Alcoholism screening was done using the CAGE test [18]. A total score of 2 or greater was considered clinically significant. Chi-square test analysis was carried out to determine the statistical significance of the association between the MMAS-8 medication adherence categories and glycemic control categories and secondly between suboptimal adherence and the different independent categorical variables. All independent variables whose p-values did not exceed 0.2 were selected for inclusion in the multivariate analysis model. Collinearity was assessed and for variables that were identified to be collinear (r ≥=0.5, using Pearson's correlation test); the variable exhibiting greater association to non-adherence namely lower p-value was selected for inclusion in the final ordinal logistic regression model. Ordinal logistic regression was used for multivariate analysis. Odds ratios (ORs), 95% confidence intervals and p-values were calculated.

## Results

A total of 290 Type 2 diabetes mellitus participants were recruited into the study. The mean age of the participants was 56.6 (SD ± 11.86) years. The mean duration of diabetes mellitus since diagnosis was 8 (SD ± 7.8) years.Socio-demographic characteristics of the participants are summarized in Table 1. The clinical profile of the study participants is presented in Table 2. Self-reported adherence to medication measured by MMAS-8 scale was low for 28.3 % (95% CI: 23.1, 33.5), medium for 26.2% (95% CI: 21.1, 31.3) and high for 45.5% (95% CI: 39.6, 51.3)of the study participants. Glycaemic control was good (HbA1c < 7%) for 107 (36.9%) and poor (HbA1c > 7%) for 183 (63.1%) of the study participants. A significant association was found between medication adherence and glycaemic control. 56.1% of participants in the high adherence category had good control compared to 24.3% and 19.6% of participants in the low and medium adherence category respectively (Table 3). Results of bivariate analysis between medication non-adherence and various independent variables are summarised in Table 4 and Table 5. Significant collinearity was observed between patient satisfaction with family members support and family members' attitude towards patient's illness and also between patient satisfaction with attending clinician and patient's overall experience at the clinic. Patient's satisfaction with family member support in regard to diabetes mellitus was selected in the first case and patient's satisfaction with attending clinician in the latter for inclusion in the final model (Table 6). Five factors emerged significantly associated with poor medication adherence in multivariate analysis; patients with duration disease between 2-10 years (OR = 2.07, CI = 1.01-4.22),ever being admitted for diabetes mellitus(OR = 2.94, CI = 1.60-5.41), dissatisfaction with family members support in regard to diabetes mellitus management (OR = 2.99, CI = 1.12-7.98), presence of a challenge to drug access(OR = 1.76, CI = 1.01-3.05) and satisfaction with attending clinician (OR = 3.58, CI = 1.36 - 9.43).

**Table 1:** Participants’ socio-demographic characteristics

Characteristic	Participants [N, (%)]
**Age(years),** **Mean (S.D.)= 56.6 (11.86)**	
**Sex**	
Male	94,(32.4)
Female	196,(67.6)
**Marital status**	
Single	31,(10.7)
Married	222,(76.6)
Divorced	14,(4.8)
Widower/Widow	23,(7.9)
**Education Level**	
No formal education	49,(16.9)
Primary	85,(29.3)
Secondary	104,(35.9)
Higher/university	52,(17.9)
**Occupation**	
Unemployed	114,(39.3)
Civil servant	60,(20.7)
Farmer	33,(11.4)
Small scale business	62,(21.4)
Casual laborer	15,(5.2)
Other	6,(2.1)
**Religion**	
Christian	283,(97.6)
Muslim	6,(2.1)
**Alcohol consumption**	
Yes	12,(4.2)
No	276,(95.8)
**Smoking habits**	
Yes	5,(1.7)
Never	231,(79.7)
Used to but stopped	54,(18.6)

**Table 2:** Participants’ clinical profiles

Characteristic	Participants [N, (%)]
**Body Mass Index ( kg/m^2^ ), Mean (S.D.)= 30.03 (7.04)**	
Normal	60,(21)
Overweight	107,(37.4)
Obese	119,(41.6)
**Glycosylated Hemoglobin(HBA1C),** Mean (S.D.)= 7.9 (1.97)	
Good (< 7%)	107,(36.9)
Poor (>7%)	183,(63.1)
**Duration of Disease(years),** Mean (S.D.) =8 (7.82)	
< 2	66,(22.8)
2 – 10	141,(48.6)
11+	83,(28.6)
**Delay in medication start following diagnosis (years),** Mean (S.D.)= 0.5 (1.76)	
Yes	38,(13.1)
No	252,(86.9)
**Type of medication**	
Oral Glucose Lowering Agents(OGLA)	129,(44.5)
Insulin	44,(15.2)
Combination therapy (Insulin + OGLA)	117,(40.3)
**Number of diabetes medications**	
1	110,(37.9)
2	149,(51.4)
3	29,(10)
4 +	2,(0.7)
**Number of diabetes –related admissions**	
0	183,(63.1)
1	71,(26.2)
2	15,(5.2)
3	9,(3.1)
4	2,(0.7)
5+	5,(1.7)
**Presence of diabetes complications**	
Yes	139,(47.9)
No	151,(52.1)
**Presence of co-morbid states**	
Yes	217,(74.8)
No	73,(25.2)

**Table 3:** Relationship between Morisky Medication Adherence Scale (MMAS-8) categories and glycaemic control

Glycaemic control	Low Adherence	Medium Adherence	High Adherence	Total
**Good (HBA1C ≤ 7%)**	26, (24.3%)	21, (19.6%)	60, (56.1%)	107, (100%)
**Poor (HBA1C >7%)**	56, (30.6%)	55, (30.1%)	72, (39.3%)	183, (100%)
***Total***	82, (28.3%)	76, (26.2%)	132, (45.5%)	

Χ^2^ =7.902 ,p-value= 0.019

**Table 4:** Relationships between patient related factors and non-adherence

Characteristic	Total	Low-Medium Adherence [N, (%)]	p-value
**Age**			0.715
18 -54	122	68,(55.7)	
55+	168	90,(53.6)	
**Sex**			0.760
Male	94	50,(53.2)	
Female	196	108,(55.1)	
**Marital status**			0.168
Married	222	116,(52.3)	
Not married	68	42,(61.8)	
**Education level**			0.236
None or Primary	134	68,(50.7)	
Secondary or Tertiary	156	90,(57.7)	
**Occupation**			0.911
Formal	122	66,(54.1)	
Informal	168	92,(54.8)	
**Alcohol consumption**			0.145
No	276	148,(53.6)	
Yes	12	9,(75.0)	
**Smoking habits**			0.357
Ever smoked	59	29,(49.2)	
Never Smoked	231	129,(55.8)	
**Satisfaction with family support?**			0.024
Satisfied	254	132,(52.0)	
Dissatisfied	33	24,(72.7)	
**Attitude of family members in regard to patient’s illness**			0.03
Positive	243	126,(51.9)	
Negative	43	30,(69.8)	
**Do Home Blood Sugar monitoring (HBSM)?**			0.506
Yes	127	66,(52.0)	
No	161	90,(55.9)	

**Table 5:** Relationships between diabetes treatment factors and non-adherence

Variable	Total	Low-Medium Adherence [N, (%)]	p-value
**Injection/insulin medication**			0.049
No	129	62,(48.1)	
Yes	161	96,(59.6)	
**Duration of Disease**			0.004
< 2 year	66	25,(37.9)	
2 – 10 years	141	88,(62.4)	
11+	83	45,(54.2)	
**Number of Diabetes Medications**			0.052
1	110	52,(47.3)	
2	149	84,(56.4)	
3+	31	22,(71.0)	
**Delay in medication start following diagnosis?**			0.011
No	252	130,(51.6)	
Yes	38	28,(73.7)	
**Ever DM related admission?**			0.004
No	183	88,(48.1)	
Yes	107	70,(65.4)	
**Presence of complication or comorbidity?**			0.193
No	42	19,(45.2)	
Yes	248	139,(56.0)	
**Frequency of DM clinic**			0.664
3 monthly or less	167	92,(55.1)	
4 monthly or more	120	63,(52.5)	
**Attendance of diabetes education session**			0.739
No	14	7,(50.0)	
Yes	275	150,(54.5)	
**Challenge in drug access?**			0.069
No	141	85,(60.3)	
Yes	126	62,(49.2)	
**Satisfaction with attending clinician?**			0.096
Satisfied	258	136,(52.7)	
Dissatisfied	29	20,(69.0)	
**Overall clinic experience**			0.104
Poor	18	10,(55.6)	
Average	75	33,(44.0)	
Good	190	111,(58.4)	

**Table 6:** Logistic regression analysis of factors associated with poor medication adherence

Variable	OR	95%CI	p-value
**Injection/insulin medication**			
No	1		
Yes	1.46	0.80-2.68	0.216
**Duration of Disease**			
< 2 year	1		
2 – 10 years	2.07	1.01-4.22	0.047
11+	0.99	0.43-2.28	0.983
**Number of Diabetes Medications**			
1	1		
2	1.16	0.63-2.12	0.631
3+	2.26	0.80-6.41	0.125
**Ever DM related admission?**			
No	1		
Yes	2.94	1.60-5.41	<0.0001
**Satisfaction with family support?**			
Satisfied	1		
Dissatisfied	2.99	1.12-7.98	0.029
**Presence of complication or comorbidity?**			
No	1		
Yes	1.85	0.84-4.06	0.125
**Satisfaction with attending clinician?**			
Satisfied	1		
Dissatisfied	3.58	1.36-9.43	0.01
**Alcohol consumption**			
No	1		
Yes	0.22	0.05-1.05	0.057
**Delay in medication start following diagnosis?**			
No	1		
Yes	2.28	0.96-5.39	0.061
**Marital status**			
Married	1		
Not married	1.20	0.60-2.37	0.609
**Challenge in drug access?**			
No	1		
Yes	1.76	1.01-3.05	0.046

## Discussion

This study found less than one in every two patients (45.5%) was fully adhering to the prescribed medications. This proportion of medication adherence is comparable to other studies done among Type 2 diabetic patients in clinic settings done in Kenya and in the Eastern Africa region [20-22]. This is a worrying trend given that the incidence of diabetes in Kenya and Africa is rising [8]. National health systems are poorly coping with the increasing burden of non-communicable diseases owing to underdeveloped public health systems and inadequate funding [23]. According to the WHO; increasing effectiveness of adherence interventions may have greater impact on the health of populations than improvements in specific medical treatments [3]. This is because however efficacious novel treatments are, when patients do not take them correctly, then the expected benefits such as averted morbidity, disability and mortality may not be realised. Indeed among Type 2 diabetic patients; a study demonstrated that all-cause hospitalisation increased by 58% and all-cause mortality increased by 81% among diabetic patients who were poorly adhering to their medications [24]. Medication non-adherence is preventable and there is great need to support patients adhere to their prescriptions. According the 2010 Ministry of Health (MOH) clinical guidelines for the management of diabetes mellitus; good control is indicated by a glycosylated hemoglobin level of less than 7% [25]. In this study one hundred and seven (36.9%) of the patients who participated in the study achieved this cut-off of blood sugar control. Studies done in Kenya and other parts of Africa have shown low levels of glycemic control ranging from 17%-38% [20, 26,27]. Chronically raised blood sugar and associated metabolic disturbances related to insufficiency in insulin production or/and insulin action is the underlying pathology in diabetes mellitus. Glycemic control is hence the ultimate objective of any diabetes mellitus therapy. Good glycaemic control among type 2 diabetes mellitus patients involves interplay of self-management measures including physical activity and diet in addition to medication adherence [3]. This can explain the discrepancy between the better medication adherence scores and poorer glycemic control outcome in this study population. Importantly these low levels of glycaemic control in Kenya and Africa in contribute to high rates of diabetes related morbidity and mortality. Globally diabetes is a leading cause of blindness, kidney failure, heart attacks, stroke and lower limb amputation [28]. This study found a significant inverse relationship between high adherence scores and lower assayed values of glycosylated haemoglobin (HbA1C). The patients with high scores in the MMAS-8 reflecting good medication adherence were also most likely to have lower and optimal glycosylated haemoglobin values. Other workers in African contexts have also demonstrated this effect [20, 21].

This finding demonstrates that medication adherence plays an important role in maintaining blood sugar levels within normal ranges. Furthermore it supports the use of quick to administer and self-reported medication adherence scales such as the MMAS-8 in busy clinical practices as a means of quickly filtering patients who are poorly adhering to medication for intensified counselling to reinforce medication adherence. Amongst factors that fuel medication non-adherence; dissatisfaction with close family members' support in regard to diabetes mellitus management emerged significantly associated with non-adherence. Poor social support has been shown in several studies to be associated with inadequate management of diabetes mellitus [27]. Most patients enrolled in this study reported that the family members played the role of encouraging and reminding them of their medication, this is important for patients suffering chronic illnesses who tend to feel isolated in their daily struggle to contain their disease. The role of a treatment supporter usually a close family member in diabetes mellitus management has often been neglected; this is in contrast with care provision for chronic communicable illness such as HIV/AIDS and tuberculosis (TB) that have long periods of medication use [29, 30]. In these two conditions patients' registration into care usually involves engaging the patient in identification of a suitable treatment supporter. The identified treatment supporter is informed of the patient's diagnosis, educated on the healthy lifestyle modifications, importance of medication adherence and clinic attendance in order that he/she may encourage the patient towards these goals. Age was not found to be significantly associated with non-adherence in this study. Whereas it has been shown that the prevalence of diabetes mellitus increases with age in Kenya [31]; medication adherence however has been shown either not be affected by the patient's age [20] or actually improve with age [32]. The majority of participants in this study was over fifty-five years of age and would likely be living with children or relatives; the protective effect of family noted above is likely to facilitate adherence with medication. In contrast younger patients who are professionally active have been shown to be more likely skip or forget their medication [33]; hence poorer adherence. Sex was also found not to be significantly associated with adherence in this study. Some studies have found that females were more likely not to adhere to medications [34, 35]. Due to the relative minority of males (32.4%) in the clinic attendance, we may have been unable to demonstrate this association. Level of education in this study was found not to be significantly associated with medication adherence. Several studies have also shown similar findings [36, 37]. The crucial aspect as regards to medication adherence as demonstrated in qualitative studies is whether a patient understands their prescribed medication [38]. This lays great importance on patient education and counseling at diagnosis and during follow-up in simple language within the patient's level of understanding. Alcohol usage has been show in other studies to be associated with medication non-adherence [39], however due to the small number of alcohol users in this study we may have been unable to demonstrate this association. Home blood sugar monitoring (HBSM) is an important part of diabetes self-management and provides the patient with an ongoing feedback on effectiveness of his/her diabetes management efforts i.e. whether blood sugar levels are within target ranges. However in our study; there was no association between the HBSM and medication adherence. This finding is consistent with findings from another study conducted in western Kenya that demonstrated low levels of blood glucose monitoring and no association with glycemic control [40]. In this study, this finding can be explained by a majority of patients lacking personal glucometers thus not practicing HBSM and for those who have glucometers not adjusting their medication based on blood sugar values obtained.

In this study; ever having been admitted for diabetes mellitus was shown to be significantly associated with of non-adherence. Physiologically poor adherence is associated with uncontrolled blood sugar levels that result in accelerated end organ damage [2]. Frequent admissions have economic impacts at the personal level and public health level. At the personal level costs accrue from direct loss in productivity and income. Cost of treatment has been implicated as a barrier in achieving medication adherence and glycemic control among Type 2 diabetes mellitus patients [41]. Almost half of diabetic patients recruited reported cost as their main challenge to medication access. In Kenya, where the bulk of health care costs are paid out of pocket (OOP); this increased expenditure can result in catastrophic impoverisation of individuals and their families especially if the breadwinner is affected [42]. At the public or national health level; increased resources need to be invested in caring for these patients including health personnel; medications and physical facilities. The estimated annual cost of diabetes in the sub-Saharan Africa region has been estimated at over 8000 United States dollars per patient [23]. Patients who have had diabetes for a period of 2 to 10 years were found in this study to have less adherence to the diabetes mellitus medication than newly diagnosed patients(duration of disease <2 years). This finding could be associated with the progressive Beta-cell failure in diabetes mellitus which results in progressive increase in the number and dosage of medications required to achieve optimal glycaemic control. Regimen complexity is also associated with an increase in medication side effects which limits the willingness of patients to take their medicines. Longer time periods between clinic appointments for these experienced patients has also been shown to contribute to poor medication adherence [34]. Disease and treatment factors such as number of diabetes mellitus medication, presence of injectable medication and presence of comorbidities and complications were on bivariate analysis significantly associated with poor adherence. However the significance in association was lost when they were placed in multivariate analysis. This could have been due to association of these factors with other factors associated with medication adherence that when controlled for were able cater for their confounding effects. These findings suggest that these factors do not predict medication adherence behaviour in this cohort of patients. Attendance of health education sessions was not significantly associated with good medication adherence. Furthermore majority of the patients had not been taught how to adjust medication based on blood sugar readings. This finding differs from a similar study conducted in Uganda which demonstrated that ever attending a health education session lowered the odds of non-adherence [37]. Whereas the classroom or group approach utilised at the clinic is effective when many patients need to be educated; the information disseminated is generalised and may not satisfy the individual needs of each patient. It has been shown that diabetic patients consider obtaining information regarding their prescribed medication as their foremost need towards medication adherence [38]. Supplementary personalised counselling and education sessions targeted at uncovering the particular adherence barriers pertaining to each patient would be of great value. Indeed studies have shown that patients receiving care from specialists who are typically busy and have less time per individual patient are less likely to adhere to their medication [33, 35]. Similarly in this study, nurses were reported to be the commonest source of diabetes health information and not the attending doctors. Satisfaction with the attending clinician emerged as a significant contributor to good medication adherence. Patients who were dissatisfied in their clinician were three times more likely to be non-adherent to their diabetic medication compared to those who were. In a similar study, patients reporting poor patient-provider communication and dismissing attachment were significantly less likely to adhere to their medication and consequently had poorer glycaemic control [43]. General dissatisfaction with the quality of health services provided at a health facility is also a recognised barrier to medication adherence in patients who received care there [20]. Dissatisfied patients are also less likely to attend follow-up clinics or attend education sessions and have little trust in the medication prescribed.

## Conclusion

In conclusion, a majority of type 2 diabetes mellitus patients have suboptimal medication adherence which is associated with poor blood sugar control. Family support, affordability of medications and good healthcare provider- patient communication are important factors in ensuring medication adherence. This study recommends that hospital management boards implement facilitated participation of family members in the diabetic patient care process, a scheme for free or subsidised medication provision as well as health provider communication trainings as foundational steps in improving medication adherence among Type 2 DM patients.

### What is known about this topic

- Adherence to medication is a common problem globally for patients with chronic illnesses including Type 2 DM;
- Medication non-adherence is associated with poor glycemic control and thus worse clinical outcomes.

### What this study adds

- Medication adherence pattern of a typical Kenyan population of Type 2 DM patients;
- The factors that drive medication non-adherence among Type 2 DM patients in Kenya, which could inform health managers in designing effective medication adherence strategies.

## Competing interests

Authors declare no competing interests.
